# Implant survival and patient satisfaction in completely edentulous patients with immediate placement of implants: a retrospective study

**DOI:** 10.1186/s12903-018-0669-1

**Published:** 2018-12-18

**Authors:** Hye-sung Kim, Han-A Cho, Young youn Kim, Hosung Shin

**Affiliations:** 1 Departments of Oral Implantology, Oral Health Science Research Center, Apple Tree Dental Hospital, 1450, Jungang-ro, Ilsanseo-gu, Goyang-si, Gyeonggi-do 10387 Republic of Korea; 2 Department of Social and Humanity in Dentistry, Wonkwang University School of Dentistry, 460 Iksan Dearo, Iksan, North Jula 54538 Republic of Korea; 3 Departments of Oral and Maxillofacial Surgery, Oral Health Science Research Center, Apple Tree Dental Hospital, 1450, Jungang-ro, Ilsanseo-gu, Goyang-si, Gyeonggi-do 10387 Republic of Korea

**Keywords:** Cumulative survival rate, Immediately placed implant, Immediate loading, Patient satisfaction

## Abstract

**Background:**

This study evaluated full-arch rehabilitation of patients with immediately placed implants in terms of the cumulative implant survival rate, risk factors for implant failure, and patient satisfaction.

**Methods:**

Time-to-event data of 52 completely edentulous jaws (370 implants) were collected using retrospective clinical chart review for the time period from 2008 to 2014. A conventional two stage approach for surgery was adopted to immediately placed implants in the maxilla, and immediate placement and immediate loading protocols for the mandible were followed. The study calculated the 7-year cumulative survival rates (CSR), and a Bayesian hierarchical Cox proportional hazard model was used to measure the effect of covariates. Patient satisfaction on chewing ability, esthetic appearance, and overall satisfaction was also measured with a face-to-face interview survey.

**Results:**

Of the total 370 implants, 194 were immediate placement. Two delayed loading maxillary implants failed within the first year, and another one failed in the second year of loading. Two failures were recorded in the first year and one in seven years for the immediate loading mandibular implants. The 1-, 5-, and 7-year CSR of the 370 implants were 0.989 (0.979, 1.000), 0.986 (0.975, 0.998), and 0.978 (0.957, 0.999), respectively. Only the length of the implant affected implant failure (*p* < 0.05); other patient characteristics, systemic diseases, implant diameter, immediate loading, and immediate placement, did not have an effect on implant failure rates. Patients reported a high degree of satisfaction regardless of their age group or length of the observation period.

**Conclusions:**

Immediately placed implant had CSR as high as delayed placed implants, and 7-year CSRs of immediate loading were not significantly different from delayed loading. The procedure also had a high degree of chewing ability, esthetic appearance, and overall satisfaction. The study results suggested that the clinical procedures applied in this study to completely edentulous patients were acceptable rehabilitation procedures.

## Background

The completely edentulous condition due to progressive loss of teeth causes esthetic and functional problems. The high degree of success of dental implants makes clinicians and patients increasingly implants for the rehabilitation of edentulous jaws. Implant protocols have been used with the aim of improving the oral and facial characteristics of completely edentulous patients: overdentures, implant-supported, and full-arch fixed implant supported prostheses. Implant-supported prostheses have been reported to be an integral part of prosthodontic treatment planning, with high success rates and low postoperative complications [1]. In addition, fixed restorations provide a feeling of similarity to natural teeth and a sense of psychological stability [2], which is a good response in completely edentulous patients [3].

Immediately placed and immediately loaded implant rehabilitation has spread in the clinical setting, but situations such as poor primary stability may compel the dentist to follow a more conventional placement and a delayed loading protocol [4]. Previous studies reported that an immediate placement and immediate loading procedure is an effective method with a reduced treatment time [2, 3] and sufficient acceptance by patients [2, 4], and it allows immediate function and an improved appearance [5]. Immediate placement and immediate loading is also associated with high survival rates and has been met with increased patient satisfaction [2, 6]. Several modalities have been reported for immediately placed implants and immediately loaded fixed prostheses and subsequent rehabilitation: 2 to 6 implants in the mandible [7, 8] and 4 to 12 implants in the maxilla [9–12]. The 3-year cumulative survival rate (CSR) of “all-on-4” was 96.2% [13], and a 5-year survival rate of 6~12 implants immediately loaded with a cross-arch fixed restoration was 95.3 to 99.29% [14]. However, the clinical outcomes may depend on the clinicians’ levels of training, experience, and skill [5] as well as proper patient selection.

In general, studies on implant survival tend to occur more than studies of patient satisfaction after implant placement. However, expressions of satisfaction can be important information for dentists to improve the quality of dental care [10]. The present study retrospectively assessed the 1-, 5-, and 7-year CSRs of immediately placed implants and the satisfaction of patients in terms of chewing ability, appearance, and overall satisfaction with full-arch rehabilitation. Furthermore, the risk factors for implant failure were analyzed.

## Methods

**Fig. 1:**
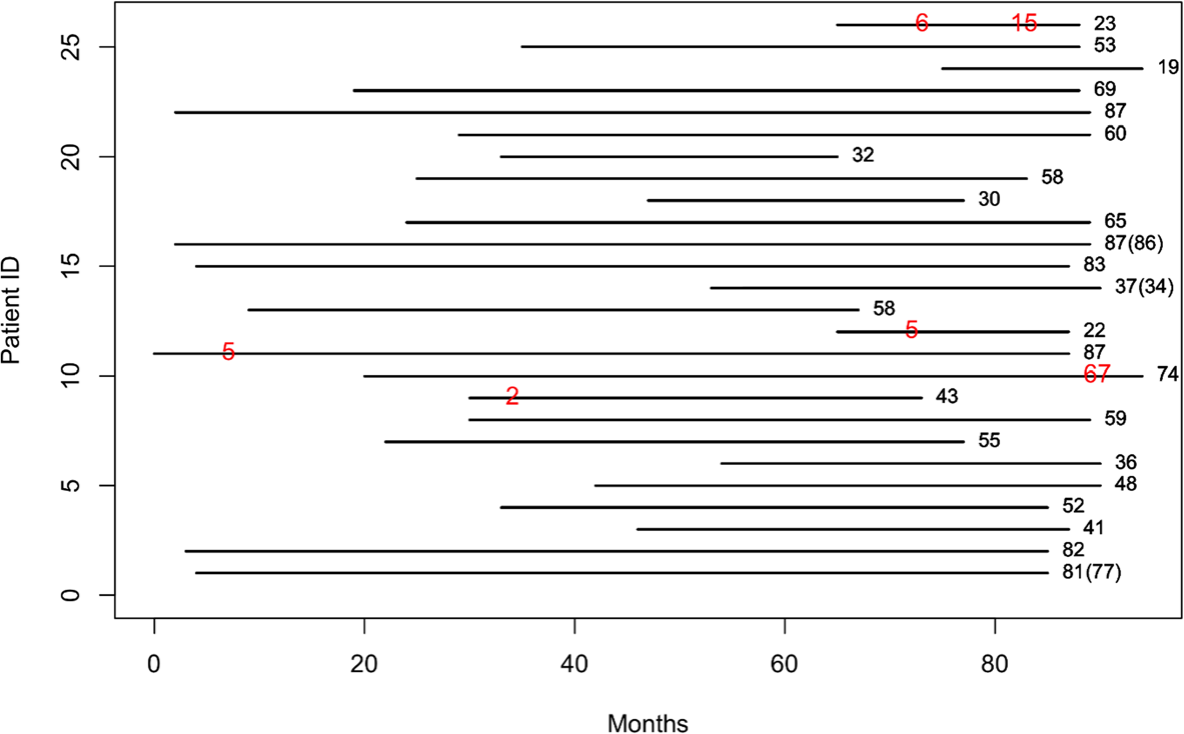
Observation periods by subject. The number at the end of the line indicates the period of follow-up, and the number in the middle of the line refers to the time of implant failure. ( ) represents the follow-up period of another jaw of the same patient

All patients provided written informed consent for participating in the study and procedures. The study was approved by our institutional review board (WKIRB-201511-BM-002). Three reviewers (two dentists and one hygienist) carefully reviewed patient charts and selected patients who met the eligibility criteria during the study period from February 2008 to May 2014. The inclusion criteria were either a poor prognosis for both the maxillary and mandibular teeth or completely edentulous jaws, and sufficient residual bone volume to receive implants at least 3.0 mm in diameter and 8.0 mm in length. Patients participating in this study were systemically controlled, and the study excluded those with uncontrolled systemic diseases. Figure 1 shows the period of follow-up and the time of the patient’s implant failure.

All subjects were evaluated using intraoral and extraoral clinical examination, including radiographs (periapical, cone-beam CT, and cephalogram). Compromised teeth with an unfavorable prognosis were extracted due to root caries or severe periodontitis. The mean number of extracted teeth per patient was six in the maxilla and seven in the mandible. Implants were placed in the fresh extraction sockets for immediate placement and in the healed sites for the conventional placement [15]. Selection criteria for immediate implant placement were atraumatic extraction with a minimum of 3–5 mm of intimate bone to implant contact [11]. The average number of immediately placed implants per patient was 3.73 (standard deviation, 2.51), and the average for immediately placed implants in the maxilla (3.96) was approximately 0.5 more than that of the mandible (3.50). Based on the anatomical locations and bone quantity, conventional delayed implants or immediately placed implants (Osstem Implant Co., Ltd., Busan, Korea and Dentium Co., Seoul, Korea) were installed in the maxilla (one in the first molar, two in the second premolars, and one in the incisor on each side) and in the mandible (one in the first molar, one in the first premolar, and one in the canine or incisor on each side). The installed implant was an internal connection type and had a diameter of approximately 3.4 to 7.0 mm in the maxilla and 3.0 to 7.0 mm in the mandible. The length of the fixture was approximately 8.0 to 14.0 mm for both the maxilla and mandible. A tapered type fixture was used in the maxilla for the purpose of increasing initial stability. The implant was placed parallel to the adjacent tooth before extraction or in as vertical a direction to the crestal bone as possible. The implant was placed under a force of 50 Newton-centimeters as the maximum torque.

**Fig. 2:**
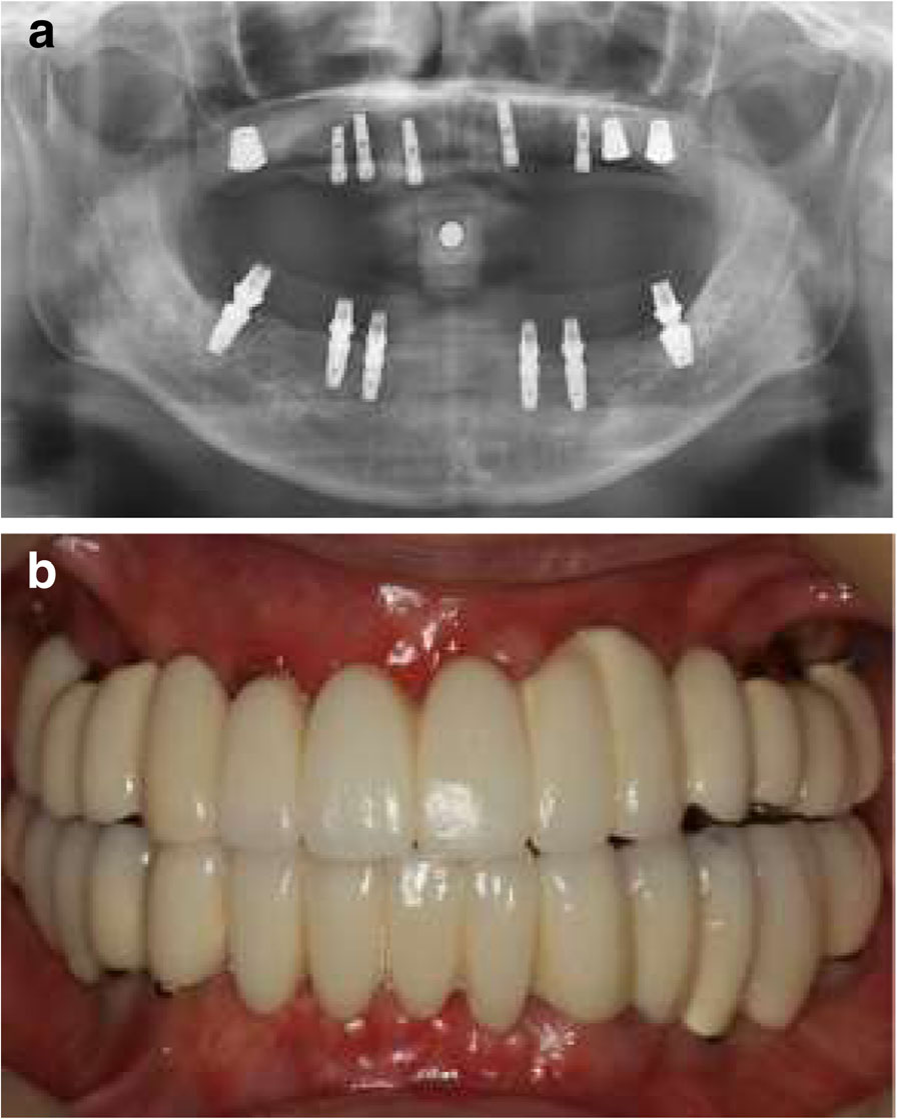
Patient after full-arch rehabilitation with immediately loaded implants. **a** Panoramic radiograph after surgery **b** Final implant supported prosthesis

Immediately after surgery, a provisional removable complete denture was placed in the maxilla, and an immediately loading fixed full-arch prosthesis was placed in the mandible. One-piece fixed prostheses were fabricated using provisional titanium cylinders abutment and a self-cured acrylic resins (SNAP; Parkell, Edgewood, NY, USA) and were cemented to ready-made abutments with TempBond (RelyX;, 3 M, St. Paul, MN, USA). For implants for immediate loading, a screw-type implant with a diameter as wide as possible was used, and a progressive loading occlusion was established [11]. After 6 months, a 2nd surgery in the maxilla was performed. Final prostheses were fabricated with an SCRP (screw cement retained prosthesis) type and were cemented (HY-Bond Polycarboxylate Cement; Shofu Dental Corp, San Marcos, CA, USA). When the SCRP type was not applicable, according to the path or position of the fixtures, a cement-retained prosthesis was set with temporary cement TempBond (Fig. 2). The metallic occlusal surface was used for the posterior maxilla to prevent fracture, and the occlusal surface of the mandibular posterior was made with ceramic.

In the current study, the criteria for implant failure were as follows: 1) failure of osseointegration, 2) the confirmed presence of uncontrolled or > 50% loss of the bony structure around the implant, and 3) the presence of pain during function or percussion after completion of the final prosthesis. These criteria were based on Albrektsson et al. [12] and the classification of Misch [13].

From December 2015 to January 2016, the patients were surveyed regarding their degree of satisfaction with the implants. The satisfaction items were chewing ability, esthetic appearance, and overall satisfaction [14, 16]. Each survey question was graded on a 5-point Likert scale: highly satisfied, satisfied, partially satisfied, unsatisfied, and highly unsatisfied. The survey was performed in subjects through a face-to-face interview by trained personnel.

### Statistical analysis

To analyze the risk factors for implant failure with time-to-event data, we used a Cox proportional hazard model. The model assumed that the time that passed before implant failure occurred might be associated with the covariates. We applied Bayesian hierarchical analysis [17, 18] to the Cox model because the sample of completely edentulous jaws was relatively small and we wanted to control for the variation of independent variables among the patients. The model assumed that patient characteristics might affect implant survival (random effects). The model is elaborated in Eq. 1:

1 $$ {\displaystyle \begin{array}{c}{h}_{ij}(t)={h}_0(t)\cdotp {e}^{\eta_{ij}}={h}_0(t)\cdotp \exp \left({\eta}_{ij}\right)\\ {}{\eta}_{ij}={b}_0+{\beta}_1{Sex}_{ij}+\sum \limits_{k=2}^3{\beta}_k{Agegp}_{kij}+{\beta}_4{Chronic}_{ij}+{\beta}_5{Dealyed}_{ij}\\ {}+{\beta}_6{Dia}_{ij}+{\beta}_7{Lth}_{ij}+{\beta}_8{Sinus}_{ij}+{\beta}_9 Immedite\_{P}_{ij}+{\mu}_j\end{array}} $$

where *i* and *j* represent an individual implant and patient, respectively. *h*_*ij*_(*t*) is the instantaneous hazard rate, in which the effects of the independent variables were considered during the finite observation period Δt, and *h*_0_(*t*) is the baseline hazard ratio. In Equation (1), *μ*_*j*_ represents the random effect and is assumed to follow a normal distribution with a mean of 0 and a variance of ${\sigma}_{\mu}^2$. The exponential value (*e*^*β*^), which is a regression coefficient of an independent variablex_i_, represents the relative hazard ratio.

Based on previous studies, the following covariates were selected for the current study: sex; age; the presence of chronic systemic diseases such as diabetes mellitus or hypertension; the implant position which is also related to immediate loading (we applied immediate loading to the mandible, and delayed loading to the maxilla); supplemental surgical procedures used (sinus lift and bone graft); the diameter and length of the implant fixture; and immediate placement. The patients were subdivided into three age groups: < 60 years, 60 to 70 years, and ≥ 70 years.

Bayesian analysis was performed using integrated nested Laplace approximation [19] with a statistical package R version 3.2.3. The equality of survivor functions between groups were tested with log-rank test, and the level of statistical significance was set at *p* < 0.05.

## Results

The total number of patients included in the study was 26 (52 jaws), the mean observation period was 55 months (minimum 19 months, maximum 87 months), and 5 patients were lost to follow-up. Of all 26 patients (52 jaws), more than two-thirds were male, and the mean age was 58.9 years. Patients aged ≥70 years (mean, 75.9 years) accounted for 23.8% of the total subjects. One-quarter of subjects had one of the chronic diseases. At the time of implant placement, approximately 12.7 teeth were extracted per patient, and more than 52% of all 370 (194 implants) were immediately placed implants. The mean number of immediately placed implants was 3.96 and 3.50 in the maxilla and in the mandible, respectively, and tended to be inverse to age in the maxilla (*p* < 0.001). Overall, an inverse relationship was shown between age and implant diameter, but there was no statistically significant relationship between the length of the implant and the age group. The mean number of installed implants was 8.11 in the maxilla and 6.12 in the mandible (Table 1).

**Table 1:** Characteristics of subjects and installed implants

Age group	Male^b^	Immediate placement					Delayed placement				
		Chronic	Lth	Dia^a^	Mx^b^	Md	Chronic	Lth^a^	Dia^b^	Mx^b^	Md^a^
< 60	0.88	0.27	10.54	4.48	5.59	4.18	0.36	9.91	4.58	2.47	1.88
60–70	0.67	0.00	10.79	4.07	1.00	1.33	0.00	10.77	3.83	7.00	4.67
> 70	0.16	0.24	10.33	4.01	0.83	2.67	0.60	10.89	3.87	7.50	3.67
Total	0.69	0.25	10.53	4.41	3.96	3.50	0.38	10.45	4.16	4.15	2.62

^a^*p* < 0.05; ^b^*p* < 0.001

“Male” represents the proportion of males by age groups

*Dia* Diameter and *Lth* length of implants are presented in millimeters (mm)

*Mx* maxilla and *Md* mandible represent a mean number of immediately placed implants and delayed placed implants in the maxilla and mandible, respectively

Six implants from 5 patients (1.6%) failed; two failed within the first year, and another one failed in the second year in the delayed loading maxillary implants. Two failures were recorded in the first year and one in seven years for the immediate loading mandibular implants. Among six failed implants, half were immediately placed implants (Table 2).

**Table 2:** Characteristics of the failed implants

Patient ID	Sex	Age group	Chronic	Immediate loading/Delayed loading	Immediate placement/Delayed placement	No. of implants per prosthesis	No. of immediately placed implants	Time of failure (months)
9	male	< 60	no	IL	IP	8	5	2
10	male	> 70	no	IL	IP	8	3	67
11	male	60–70	no	IL	DP	8	6	5
12	male	< 60	no	DL	IP	6	6	5
26	male	< 60	no	DL	DP	6	3	6
26	male	< 60	no	DL	DP	6	3	15

*IL* immediate loading, *DL* delayed loading, *IP* immediate placement, *DP* delayed placement

**Fig. 3:**
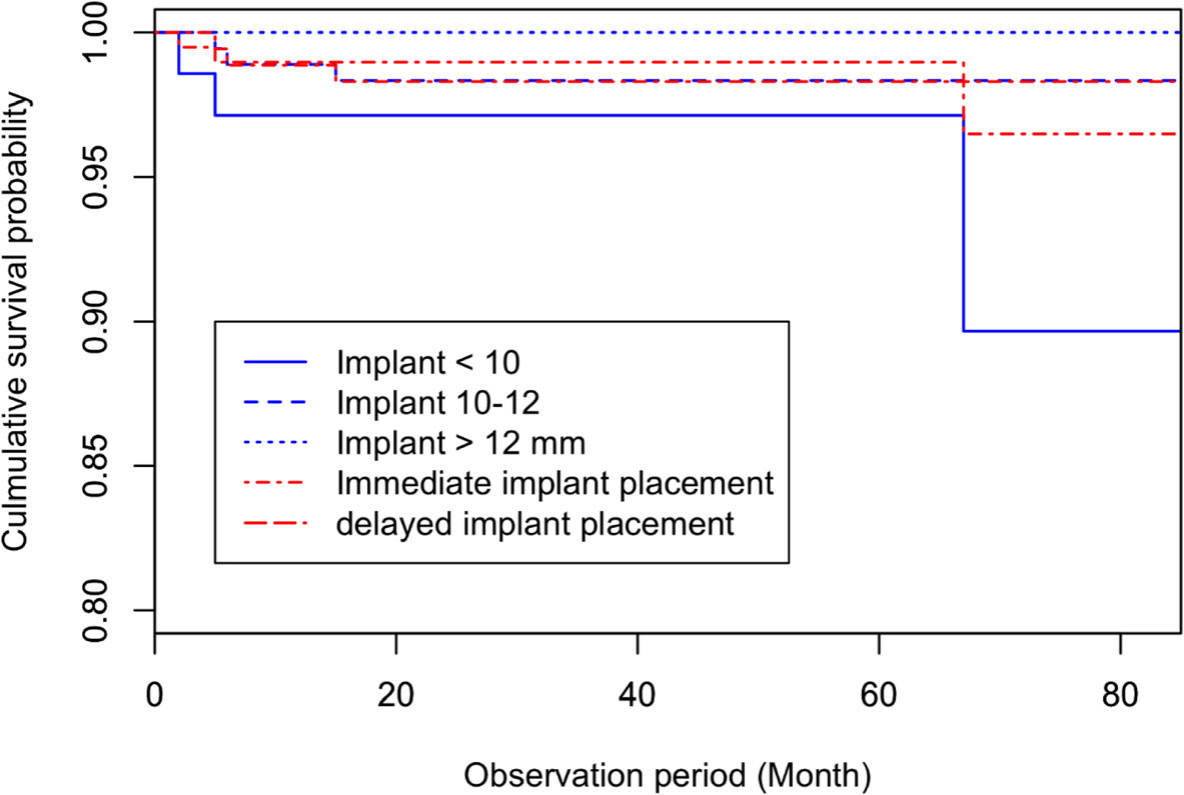
Implant survival rates by implant length and types of implant

The 1-, 3-, 5-, and 7-year CSRs were 0.989, 0.986, 0.986, and 0.978, respectively (Table 3). The 7-year CSR of immediate loading was 0.968 and 0.986 of delayed loading, and was not statistically different (*p* = 0.72). Differences in the CSR depended on the length of the implant; a longer implant was significantly associated with a higher survival rate (*p* < 0.01). The 7-year CSRs for implants of > 12, 10 to 12, and < 10 mm were 1.000, 0.990, and 0.962, respectively. The 7-year survival rates were not significantly different between the immediate placement and delayed conventional placement (Fig. 3).

**Table 3:** Cumulative survival risks by observation time

Classification	Time (month)	n.risk^a^	Event	CSR	*p*-value	95% CI	
Total	12	366	4	0.989	–	(0.979	1.000)
	36	289	1	0.986		(0.975	0.998)
	60	142	0	0.986		(0.975	0.998)
	72	97	1	0.978		(0.957	0.999)
	84	41	0	0.978		(0.957	0.999)
Loading type							
Immediate	12	157	2	0.987	0.72	(0.970	1.000)
	36	122	0	0.987		(0.970	1.000)
	60	64	0	0.987		(0.970	1.000)
	72	44	1	0.968		(0.928	1.000)
	84	19	0	0.968		(0.928	1.000)
Delayed	12	209	2	0.991		(0.978	1.000)
	36	167	1	0.986		(0.970	1.000)
	60	78	0	0.986		(0.970	1.000)
	72	53	0	0.986		(0.970	1.000)
	84	22	0	0.986		(0.970	1.000)
Implant length							
< 12 mm	12	38	0	1.000	> 0.01	(1.000	1.000)
	36	33	0	1.000		(1.000	1.000)
	60	21	0	1.000		(1.000	1.000)
	72	20	0	1.000		(1.000	1.000)
	84	12	0	1.000		(1.000	1.000)
10–12 mm	12	103	0	1.000		(1.000	1.000)
	36	87	1	0.990		(0.972	1.000)
	60	50	0	0.990		(0.972	1.000)
	72	33	0	0.990		(0.972	1.000)
	84	13	0	0.990		(0.972	1.000)
> 10 mm	12	225	4	0.983		(0.966	1.000)
	36	169	0	0.983		(0.966	1.000)
	60	71	0	0.983		(0.966	1.000)
	72	44	1	0.962		(0.919	1.000)
	84	16	0	0.962		(0.919	1.000)

^a^n.risk refers to the number of implants at risk, *CSR* cumulative survival rate

Table 4 shows the relative hazard ratios of factors affecting the failure rates of the 370 implants. Immediate placement and immediate loading were not risk factors for implant failure in these subjects. Of all the risk factors, however, only the length of the implant demonstrated statistical significance. The length of the implant was negatively correlated with the probability of implant failure. The variance of *μ*_*j*_ was relatively small due to the large value of precision (τ) of *μ*_*j*_.

**Table 4:** Results of Cox proportional hazard model

Variables	Coefficient	SD	Exp (Coef)	95% Credible interval	
Sex	−7.44	11.22	0.00	−33.90	8.63
Age 60–70	−0.76	1.33	0.47	−3.59	1.63
Age > 70	−0.40	0.66	0.67	−1.59	0.98
Chronic	−6.29	11.76	0.00	−34.01	10.52
Delayed loading	−1.27	1.19	0.28	−3.85	0.81
Diameter	−1.02	0.78	0.36	−2.67	0.41
Length	−0.73	0.38	0.48	−1.53	− 0.03
Sinus lift	2.64	1.45	13.98	− 0.00	5.69
Immediate placement	0.55	0.93	1.73	− 1.28	2.38

*SD* standard deviation, *Exp Coef* Exponential of Coefficient

The patients reported a high degree of satisfaction irrespective of their age group or length of time following implant placement (Table 5). Of all 26 patients, 19 participated in the satisfaction questionnaire (2 stayed abroad (patient ID-16 and 23), 4 were lost to follow-up, and 1 died) and rated their overall satisfaction of full-arch implant rehabilitation as “highly satisfied.” Most of them (above 95%) also scored both their chewing ability and esthetic outcome as above “satisfied,” but chewing ability received a higher score than esthetic satisfaction. None of these responses were statistically significant with respect to the age group or length of the observation period.

**Table 5:** Patient satisfaction with the implants

	Chewing			Esthetic			Overall satisfaction	
	HS	SA	Others (1)	HS	SA	Others (1)	HS	Others (2)
Age (years)								
< 50	7 (100.0)	0 (0.0)	0 (0.0)	6 (85.7)	1 (14.3)	0 (0.0)	7 (100)	0 (0.0)
50–60	9 (100.0)	0 (0.0)	0 (0.0)	4 (44.4)	4 (44.4)	1 (11.1)	9 (100)	0 (0.0)
> 60	2 (67.7)	1 (33.3)	0 (0.0)	2 (66.7)	1 (33.3)	0 (0.0)	3 (100)	0 (0.0)
Observation period (months)								
< 36	11 (100.0)	0 (0.0)	0 (0.0)	9 (81.8)	1 (9.1)	1 (9.1)	11 (100)	0 (0.0)
37–59	5 (100.0)	0 (0.0)	0 (0.0)	2 (40.0)	3 (60.0)	0 (0.0)	5 (100)	0 (0.0)
> 60	2 (66.7)	1 (33.3)	0 (0.0)	1 (33.3)	2 (66.7)	0 (0.0)	3 (100)	0 (0.0)

Satisfaction was measured with a 5-point Likert scale; *HS* highly satisfied, *SA* satisfied, and others (1) include partially satisfied, unsatisfied, and highly unsatisfied, and others (2) include satisfied, partially satisfied, unsatisfied, and highly unsatisfied

Data are presented as frequency (%)

## Discussion

In this study, we evaluated patient satisfaction and factors affecting implant failure of immediate placement and immediate loading rehabilitation in completely edentulous jaws. Bayesian Cox regression analysis demonstrated that only the length of the implant affected implant failure rates. Furthermore, the survey of patient satisfaction for 1.5 to 7.0 years after implant placement revealed a high degree of satisfaction, and no statistically significant differences in patient satisfaction occurred over time.

The survival rates of the implants in completely edentulous jaws in this study were similar to those reported in previous studies. In one study of the edentulous maxilla with the full arch modality supported by four to eight implants, the 3- to 10-year survival rate was approximately 95.5 to 97.9% [20]. Clinically, the number of implants required for restoration of a fixed prosthesis in edentulous patients is 6 to 8 in the maxilla and 4 to 10 in the mandible [20]. Several studies of Korean patients have also reported that a 6 to 8 in the maxilla and 4 to 10 in the mandible protocol was accepted with high esthetic and functional satisfaction [21, 22]. Del Fabbro et al. [23] reviewed the survival rates of immediately loaded implants and reported that the average number of implants needed for rehabilitation was 4.54 for the mandible and 7.82 for the maxilla on average, and the associated survival rates were 97.25 and 98.24%, respectively. Seo et al. [24] reported a 3-year CSR of 98.6% for 6 to 10 immediately loaded implants in 17 edentulous mandibles, indicating that placement of 6 to 10 immediately loaded implants was a stable procedure in edentulous Korean patients. These findings demonstrate the acceptable reliability of implant rehabilitation in the present study.

The protocol applied in this study was an immediate placement and immediate loading procedure in the mandible and immediate placement in the maxilla. A study of the effectiveness of the immediate placement and immediate loading protocol in mandibular edentulous patients with more than 5 implants showed a 98% CSR for fixtures and a 100% CSR for the prosthesis [25]. According to a 5-year retrospective study of 6–12 immediately placed implants and immediately loaded fixed prostheses with maxillary edentulous patients, the CSR for less than 10 implants was 99.29 and 96.30% for more than 10 implants [9]. In addition, the number of implants for an immediately loaded protocol with cross-arch fixed dental prostheses was 2 to 6 [6, 8, 26–29] in the mandible and 4 to 12 in the maxilla [8, 9, 27, 28, 30, 31]. Testori et al. [15] reported that the CSR difference of immediate loading and delayed loading was not statistically significant, indicating that the advantage of immediate loading was not significantly different from delayed loading. The above results are in agreement with the findings of the present study, which means that immediate loading did not adversely affect the 7-year CSR for implant survival.

In the present study, sex and age had no effect on implant failure. The findings of previous studies examining the effects of sex and age on implant survival were somewhat controversial. Degidi et al. [9] reported that the probability of prosthesis failure was lower in women, and older patients demonstrated an increased probability of implant failure. On the other hand, Krebs et al. [32] reported a CSR of 93.7% in women, which was higher than the CSR of 92.8% in men at 204 months. The authors also reported that no statistically significant differences between age groups were detected within the first year. French et al. [33] also reported that the possibility of treatment failure was higher in men. However, Busenlechner et al. [34] stated that there was no evidence that older age was a risk factor for implant survival.

Many studies have suggested that systemic conditions might be related to treatment failure after implant placement. Autoimmune diseases are associated with failure [33], and early implant failure is associated with osteoporosis [35]. Chen et al. [36] performed a meta-analysis and demonstrated that the presence of diabetes mellitus and osteoporosis were not risk factors. In the present study, patients with hypertension and type 2 diabetes mellitus demonstrated similar results as patients in previous studies [37], and this might be because they were systemically controlled.

Physical properties, such as the diameter and length of the implants, also affect the survival of the implant. Krebs et al. [32] reported that there was no significant difference in the CSR for implants with a diameter of 4.5, 5.5, or 7.0 mm. Degidi et al. [9] noted, however, that the rate of failure was higher for implants with a larger diameter (> 5.25 mm). The length of the implant is a key element in achieving the maximum strength of primary stability [30]. French et al. [33] reported that the 7-year CSR of implants with lengths of 6, 8, and ≥ 10 mm were 96, 98, and 99%, respectively. Meanwhile, in a study in which the survival rate of 980 implants was analyzed [38], the length of the implant had no significant correlation with the implant survival rate. In the present study, the implant diameter did not affect implant survival, but the implant length demonstrated a statistically significant correlation with the survival rate. The probable reasons might be that increasing implant length plays an important role in reducing bone stress and improving implant stability in poor-quality bones [39]. In addition, more is the implant length, greater is the primary stability of the implant [40].

Patient satisfaction with clinical outcome is a critical factor associated with an improved quality of treatment [10]. Patient-centered measures such as patient satisfaction should be simultaneously evaluated with objective evidence (survival rate, bone height, etc.) in implant studies [36, 37]. However, studies of implant survival have mainly focused on patient characteristics or the physical properties of the implant, and most have been based on clinical evidence demonstrating implant survival. A study that evaluated patient satisfaction using the Oral Health Impact Profile 49 reported a significant degree of patient satisfaction with implant treatment [41]. The authors of that study claimed that implant therapy improved the psychological well-being of patients, improved functionality, and enhanced the general health status. In the present study, there was a high degree of patient satisfaction regardless of patient age or the length of the period following implant placement, which is also consistent with previous studies [14, 16, 31].

There are some limitations. First, this study had insufficient clinical data (e.g., marginal bone levels etc.) due to the retrospective approach based on clinical data. Second, this study implies the possibility of selection bias because the sample was convenient and small. This might be considered insufficient to generalize the findings. Nonetheless, the CRS over a seven-year period and the Cox regression results were substantial. The benefits of the study were the combination of clinical results and patient-centered outcome associated with immediately placed implants with full-arch rehabilitation and the relatively long observation periods. Use of the Bayesian hierarchical model also improved the statistical power in the present study. However, to improve the reliability and validity of the findings, the study needed to include the key clinical measures and to increase the number of study participants.

## Conclusions

The methods applied in this study might be considered an alternative procedure by which a sufficient level of reliability can be accepted based on the maximum 7-year CSR of 97.9%. It also demonstrates that the risk of implant failure was associated with only the length of implant. A high degree of patient satisfaction, in terms of chewing ability and esthetics, was achieved with either immediate or delayed loading protocols.

## Abbreviations

CSR: Cumulative survival rate
Exp: Exponential
HS: Highly satisfied
n.risk: Number of implants at risk
SA: Satisfied
SD: Standard deviation
